# Effects of acute intermittent hypoxia on corticospinal excitability within the primary motor cortex

**DOI:** 10.1007/s00421-022-04982-8

**Published:** 2022-06-25

**Authors:** Shivani Radia, Ann-Maree Vallence, Hakuei Fujiyama, Rose Fitzpatrick, Sarah Etherington, Brendan R. Scott, Olivier Girard

**Affiliations:** 1 Discipline of Medical, Molecular and Forensic Sciences, Murdoch University, Perth, Australia; 2 Centre for Healthy Ageing, Murdoch University, Perth, Australia; 3 Discipline of Psychology, Murdoch University, Perth, Australia; 4 Murdoch Applied Sports Science Laboratory, Discipline of Exercise Science, Murdoch University, Perth, Australia; 5 School of Human Sciences (Sport Science, Exercise and Health), University of Western Australia, Perth, Australia; 6 Centre for Molecular Medicine and Innovative Therapeutics, Murdoch University, Perth, Australia

**Keywords:** Acute intermittent hypoxia, Corticospinal excitability, Transcranial magnetic stimulation, Primary motor cortex, Intracortical inhibition

## Abstract

**Purpose:**

Acute intermittent hypoxia (AIH) is a safe and non-invasive treatment approach that uses brief, repetitive periods of breathing reduced oxygen air alternated with normoxia. While AIH is known to affect spinal circuit excitability, the effects of AIH on cortical excitability remain largely unknown. We investigated the effects of AIH on cortical excitability within the primary motor cortex.

**Methods:**

Eleven healthy, right-handed participants completed two testing sessions: (1) AIH (comprising 3 min in hypoxia [fraction of inspired oxygen ~ 10%] and 2 min in normoxia repeated over five cycles) and (2) normoxia (NOR) (equivalent duration to AIH). Single- and paired-pulse transcranial magnetic stimulations were delivered to the primary motor cortex, before and 0, 25, and 50 min after AIH and normoxia.

**Results:**

The mean nadir in arterial oxygen saturation was lower (*p* < 0.001) during the cycles of AIH (82.5 ± 4.9%) than NOR (97.8 ± 0.6%). There was no significant difference in corticospinal excitability, intracortical facilitation, or intracortical inhibition between AIH and normoxia conditions at any time point (all *p* > 0.05). There was no association between arterial oxygen saturation and changes in corticospinal excitability after AIH (*r* = 0.05, *p* = 0.87).

**Conclusion:**

Overall, AIH did not modify either corticospinal excitability or excitability of intracortical facilitatory and inhibitory circuits within the primary motor cortex. Future research should explore whether a more severe or individualised AIH dose would induce consistent, measurable changes in corticospinal excitability.

**Supplementary Information:**

The online version contains supplementary material available at 10.1007/s00421-022-04982-8.

## Introduction

Neurodegenerative disorders represent one of the leading causes of mortality, comprising approximately 12% of deaths globally (Tamburin et al. 2019). Traditional pharmacological and physical activity-based therapies have limited efficacy in treating neurodegenerative disorders. Neuroplasticity refers to the ability of the central nervous system (CNS) to adapt in response to intrinsic and extrinsic stimuli and forms the basis for functional alterations (Cramer et al. 2011). Neuroplasticity is important for the development and recovery of essential functions, including motor function, following injury or neurodegeneration, e.g., spinal cord injury and stroke (Khan et al. 2017). Recently, conditioning of the CNS using low oxygen exposure has gained popularity as a potential non-pharmacological neurotherapeutic solution to treat or improve symptoms associated with movement disorders, likely via inducing neuroplasticity (Tamburin et al. 2019).

Acute intermittent hypoxia (AIH) refers to brief, repetitive periods of breathing a reduced (e.g., 10%) fraction of inspired oxygen (FiO_2_), alternated with ambient room air or normoxia (i.e., FiO_2_ 21%) (Navarrete-Opazo and Mitchell 2014; Baillieul et al. 2017). Applications of AIH range from performance enhancement of elite athletes to a therapeutic target in patients suffering from various cardio-metabolic and neurological disorders (Hurtado, 1960; Morton and Cable 2005; Dale et al. 2014; Gonzalez-Rothi et al. 2015b). In animals, AIH can trigger endogenous mechanisms upregulating the expression of various neurotransmitters and neurotrophic factors (Prabhakar, 2001; Gangwar et al. 2020). Several AIH studies investigating changes in the human CNS have focussed on patients with spinal cord injury and demonstrated chronic benefits for walking speed and endurance in individuals with incomplete lesions (Lovett-Barr et al. 2012; Oudega and Perez 2012; Sandhu et al. 2021; Sutor et al. 2021; Tan et al. 2021) with recent studies focusing on changes in corticospinal excitability following AIH (Christiansen et al. 2018, 2021; Welch et al. 2021). However, the acute effects of AIH on the CNS, specifically regarding corticospinal function, remain largely unknown.

Transcranial magnetic stimulation (TMS) is a safe and non-invasive brain stimulation technique, which can be used to assess the net level of cortical excitability and inhibitory circuit activity (Merton and Morton 1980; Barker et al. 1985; Hallett 2000). An electric pulse is delivered through a hand-held coil to generate a magnetic field, which induces an electrical current in the underlying brain tissue (Di Lazzaro et al. 2012; Di Lazzaro and Ziemann 2013). If a single-pulse TMS is delivered to the primary motor cortex (M1) with sufficient intensity, it depolarizes underlying neuronal populations. The physiological effect of TMS over M1 can be observed by measuring the motor-evoked potential (MEP) with surface electromyography (EMG). MEP amplitude reflects the extent of corticospinal excitability (Hallett 2007); a larger MEP amplitude generally indicates greater corticospinal excitability reflecting the involvement of a large number of activated neurons (Ridding and Rothwell 2007). Changes in corticospinal excitability (i.e., MEP amplitude) are thought to reflect the changes in the excitability of corticospinal and spinal motor neurons and have been widely used as a marker of neuroplasticity (Merton and Morton 1980; Hallett 2000, 2007; Rossini et al. 2015). Paired-pulse TMS protocols have similarly been used to probe neuroplastic changes in the CNS, using different stimulation protocols to distinguish between changes in facilitatory and inhibitory circuit activity (Suppa, 2008). These protocols involve delivery of two stimuli using a single TMS coil: a conditioning stimulus followed by a test stimulus across varying interstimulus intervals (ISIs, the time delay between the first and second stimuli) (Di Lazzaro and Ziemann 2013). The combination of single- and paired-pulse TMS protocols can characterise and quantify the impact of an acute condition such as AIH on motor cortex function and corticospinal excitability.

To our knowledge, only one study has evaluated how AIH for 30 min acutely modulates corticospinal excitability in healthy young adults (Christiansen et al. 2018). Single-pulse MEP amplitude and cervicomedullary MEPs increased by ~ 40% for at least 75 min following AIH. In the same study, short-interval intracortical inhibition (SICI) and intracortical facilitation (ICF), measured with paired-pulse TMS and F-waves, measured by electrical stimulation, were unchanged. The increase in MEP amplitude elicited by TMS and electrical stimulation (sub-cortical in origin) suggests that AIH can increase corticospinal excitability without modulating cortical processing, indicating that these changes are likely of a sub-cortical origin and primarily related to corticospinal synaptic plasticity (Christiansen et al. 2018). While these results have promising clinical implications, the effects of AIH on corticospinal excitability need to be further investigated by considering additional TMS measures that were not assessed by Christiansen et al. (2018). Specifically, examining the input/output curve, a more comprehensive measure that provides an indication of the states of corticospinal excitability is warranted (Carson et al. 2013). Additionally, measuring short-interval intracortical facilitation (SICF) would provide indications of the neuroplasticity of intracortical facilitatory circuits with AIH (Doeltgen and Ridding 2011).

Therefore, this study aimed to examine corticospinal excitability and the excitability of excitatory and inhibitory intracortical circuits (MEP amplitude, input/output curve, SICI, ICF, and SICF) using single- and paired-pulse TMS protocols, immediately, 25 and 50 min after exposure to AIH and normoxia. We hypothesised that there would be (1) an increase in MEP amplitude and input/output curves will show overall potentiation after AIH but not in normoxia, and (2) no changes in intracortical facilitation or inhibition (i.e., SICI, SICF, and ICF) after AIH or normoxia.

## Methods

### Participants

Nineteen right-handed participants (Edinburgh Handedness Inventory score > 40; Oldfield 1971) completed the study. All participants were screened with a TMS Safety Screen and excluded if there were any contraindications to TMS based on established international guidelines (Rossi et al. 2011, 2021), if they were taking medications acting on the CNS, or if they had any exposure to terrestrial altitude/intermittent hypoxia in the last 3–6 months. To include data on the participants who demonstrated a considerable response to the entire duration of the hypoxic protocol, a decrease of at least 3% in relative arterial oxygen saturation (SpO_2_, i.e., mean SpO_2_ across 25-min hypoxic session relative to that for normoxic session) was required, and only those who met this criterion were included in the analyses (6 participants excluded). All TMS trials were screened for background EMG activity in the 50 ms preceding TMS, and only those participants who did not show EMG activity > 0.01 mV during this time period were included in the analyses (two participants excluded). Following these exclusions, analysis was conducted on results from 11 participants (7 females; age: 29 ± 8 years; age range: 21–54 years). Prior to commencing the study, all participants provided written, informed consent in accordance with the Declaration of Helsinki. This study was approved by the Murdoch University Human Research Ethics Committee (2019/033).

### Experimental design

**Fig. 1:**
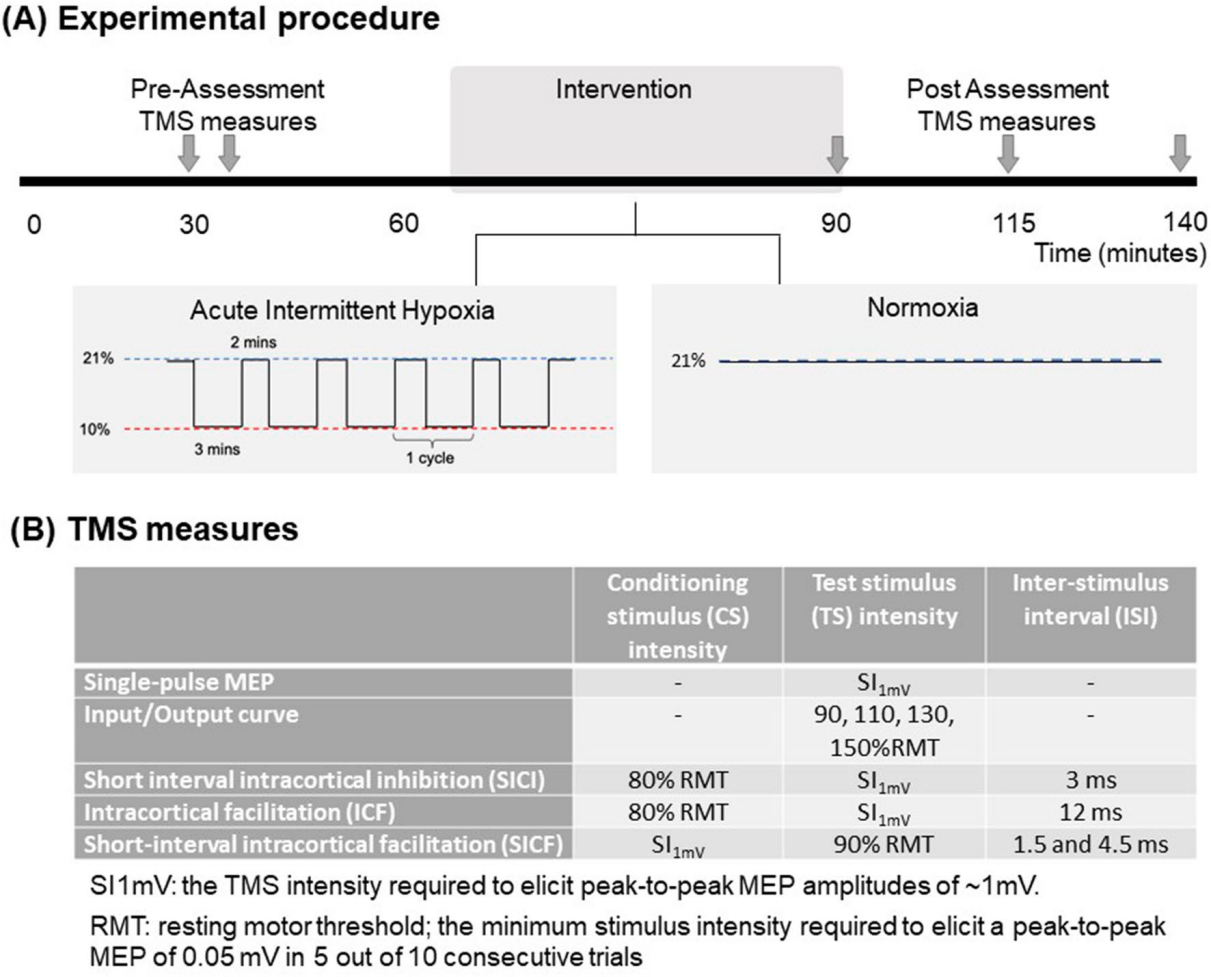
Experimental procedure timeline and TMS measures. Experimental procedure: oxygen levels and neurophysiological data were recorded at two time-points before (pre), and at three time-points after (post) the intervention (shown by arrows) (**A**). Intervention in each session comprised either 25 min of AIH (over 5 cycles) or 25 min of normoxia. Stimulation parameters for the TMS measures collected at each of the time-points before and after the intervention (**B**)

On separate days (at least 3 days apart), participants underwent two laboratory sessions: AIH and normoxic sham (NOR). The order of the session was pseudo-randomised and participants were blinded to the experimental condition. In each session, TMS was used to elicit neurophysiological responses (measured via surface electromyography; EMG) before (pre) and 0, 25, and 50 min after the delivery of the condition (Fig. 1).

Hypoxia was delivered through a mask connected to a hypoxic generator (Altitrainer, SMTEC SA, Nyon, Switzerland). The mask covered the participant’s nose and mouth and sealed around the cheeks and under the chin to prevent leaks through the mask. The AIH protocol consisted of 3 min in hypoxia (FiO_2_ ~ 10%) followed by 2 min in normoxia (FiO_2_ ~ 21%), repeated five times (total of 15-min hypoxia and 10-min normoxia). The NOR session consisted of breathing normoxic air (FiO_2_ ~ 21%) for 25 min. The hypoxic generator was hidden from participants’ view throughout the experimental sessions. For NOR, the hypoxic generator was on and set at a simulated altitude of 100 m, and pre-recorded sounds of the hypoxic generator were played to provide background noise and improve condition blinding. In the AIH session, the mask was removed during periods of normoxia; and mask application was kept the same as the AIH session throughout the NOR session. Safe hypoxic exposure in this body of literature is believed to range between 9 and 16% (Navarrete-Opazo and Mitchell 2014). We used a FiO_2_ of 10% in this study, because the hypoxic generator could not accommodate FiO_2_ values less than 10%. While this is slightly less severe than 9.4% FiO_2_ used by Christiansen et al. (2018), we attempted to counteract this by applying a longer hypoxic duration of 3 min (as opposed to 1 min used by Christiansen et al. 2018). Consequently, participants could reach lower SpO_2_ levels than they may have done with only 1 min of exposure.

### Arterial oxygen saturation levels

The SpO_2_ was recorded every 20-s during experimental trials using a pulse oximeter positioned on the left index finger (Rossmax SB100, Switzerland; averages data over 4-s epochs). The nadir in SpO_2_ of each cycle was determined as the minimum of these data. The SpO_2_ data were also analysed as 1-min mean values for the duration of the 25-min condition, as well as the mean value across the cycles (i.e., mean of the five 3-min cycles).

### Electromyographic recordings

Surface EMG activity was recorded from the first dorsal interosseus (FDI) muscle of the right hand through surface electrodes (Ag–AgCl). The skin was cleaned with ethanol and gauze before the active electrode was placed over the muscle belly and the reference electrode was placed on the metacarpophalangeal joint. A grounding electrode was placed on the medial epicondyle. The EMG data were amplified (× 1000) and band-pass filtered (20–1000 Hz) using a CED 1902 amplifier (Cambridge Electronic Design, Cambridge, UK), and digitised at a sampling rate of 5000 Hz using a CED 1401 analogue-to-digital converter (Cambridge Electronic Design, Cambridge, UK). All EMG recordings were taken during resting state with participants asked to remain still, quiet and alert.

### Transcranial magnetic stimulation

TMS was applied to the left M1 using a 90 mm figure-of-eight coil connected to a BiStim module that connected two MagStim 200^2^ Bistim magnetic stimulators (Magstim Co., Whitland, UK). The coil was held tangentially, at a 45° angle (to the sagittal plane) over the scalp to induce a posterior–anterior current flow in the underlying brain tissue.

In each experimental session, the optimal site of stimulation for eliciting an MEP in the right FDI was determined. The optimal site was defined as the scalp site that elicited the largest and most consistent MEPs (Rossini et al. 2015). The optimal site was marked on the scalp at the start of each session and used for all subsequent stimulations in the session. In each session, two TMS intensities were determined: (1) resting motor threshold (RMT), and (2) the 1 mV stimulus intensity (SI1mV). RMT is defined as the lowest stimulation intensity (as a percentage of maximal machine output) that produced MEPs of ≥ 0.05 mV peak-to-peak amplitude at rest, in at least 5 out of 10 consecutive trials (Rothwell 1997; Rossini et al. 2015). SI1mV is defined as the stimulation intensity (as a percentage of maximum machine output) required to evoke a peak-to-peak MEP of ~ 1 mV.

#### Transcranial magnetic stimulation outcome measures

During experimental trials, the input/output curve, SICI, ICF, and SICF were obtained before (pre) and at three time-points after (0, 25, and 50 min post) the condition (AIH or NOR). To ensure a stable baseline, two blocks of all TMS measures were conducted. The order of these measures was pseudo-randomised across participants and across sessions.

##### Input–output curve

The excitability of the corticospinal tract was assessed by obtaining input/output curves. Single-pulse TMS at intensities corresponding to 90, 110, 130, and 150% of each individual’s RMT were delivered (Devanne et al. 1997; Rossini et al. 2015). Ten trials were delivered at each stimulus intensity (total of 40 trials per block). The order of stimulus intensities was randomised and the inter-trial interval was set at 5 s (± 20% jitter).

##### Short-interval intracortical inhibition (SICI) and intracortical facilitation (ICF)

Single- and paired-pulse TMS was delivered to measure SICI and ICF. The paired-pulse protocol comprised a subthreshold conditioning stimulus set at 80% of RMT and a test stimulus set at SI1mV intensity, separated by an ISI of 3 ms for SICI (Kujirai et al. 1993) and 12 ms for ICF (Ziemann et al. 1998b). Each block comprised 15 paired-pulse trials targeting SICI, 15 paired-pulse trials targeting ICF, and 10 single-pulse trials (total 40 trials per block). The order of trials was pseudo-randomised and the inter-trial interval was set at 5-s (± 20% jitter).

##### Short-interval intracortical facilitation (SICF)

Single- and paired-pulse TMS was delivered to measure SICF. The MEP elicited by TMS is the result of a complex descending volley of electrical activity comprising a direct wave (D-wave) and several indirect waves (I-waves): paired-pulse TMS can be used to probe early I-wave and late I-wave circuit excitability by varying the ISI (Tokimura et al. 1996; Ziemann et al. 1998a; Chen and Garg 2000). The paired-pulse protocol for SICF comprised a conditioning stimulus set at SI1mV intensity and a subthreshold test stimulus set at 90% of RMT separated by ISIs of 1.5 and 4.5 ms. Each block comprised 15 paired-pulse trials with an ISI of 1.5 ms, 15 paired-pulse trials with an ISI of 4.5 ms, and 10 single-pulse trials (total 40 trials per block). The order of trials was randomised and the inter-trial interval was set at 5 s (± 20% jitter).

### Data processing

#### Arterial oxygen saturation data

For correlation analysis, SpO_2_ data from the hypoxia session were normalised by presenting as a ratio of the SpO_2_ values for the normoxia session for each participant.

#### Transcranial magnetic stimulation data

Single-pulse MEP amplitudes obtained during SICI, ICF, and SICF measurements (total 20 single-pulse trials) were averaged at each time point (pre, 0, 25, and 50 min post-intervention). The mean paired-pulse MEP amplitude (i.e., conditioned MEP) was expressed as a ratio of the mean single-pulse MEP amplitude at each time point. Ratios < 1 reflect inhibition and ratios > 1 reflect facilitation. Neurophysiological measures were also normalised by expressing the post-time point measures (i.e., the average of 0, 25, and 50 min post-intervention) as a percentage of the pre measure.

### Statistical analyses

Significance for all statistical analyses was set at *p* < 0.05. Group data are all presented as the mean (M) ± standard deviation (SD). All analyses were completed using IBM SPSS version 24 (IBM Corp, Armonk, NY).

For SpO_2_ data, a two-way repeated-measures ANOVA was performed on SpO_2_ data to determine whether levels differed between cycles (10% and 21%) and sessions (AIH and NOR). Separate ANOVAs were performed on the average and nadir SpO_2_ data.

For baseline TMS measures, repeated-measures ANOVAs were used to compare the single-pulse MEP amplitudes, SICI, ICF, and SICF between the two Pre-measurement blocks in the two sessions (see Supplementary Table 1). For single-pulse MEP amplitude, SICI and ICF, the ANOVAs had within-subjects factors of Pre-Measurement Block (two levels: Pre-1 and Pre-2) and Session (two levels: AIH and NOR). For SICF, two-way ANOVAs were performed separately for the two ISIs (1.5 ms and 4.5 ms) with the within-subjects factors of Pre-Measurement Block (two levels: Pre-1 and Pre-2) and Session (two levels: AIH and NOR). As there were no significant differences between the two Pre-measurement blocks for any of the measures, the two Pre-measurement blocks for each of the measures were averaged and used for all further analyses. A repeated-measures ANOVA was used to compare the Pre input/output curves between the two sessions, with within-subjects factors of Stimulus Intensity (four levels: 90% RMT, 110% RMT, 130% RMT, and 150% RMT) and Session (two levels: AIH and NOR). There were no significant differences in the Pre input/output curves between the AIH and normoxia sessions (see Supplementary Table 2).

To analyse the effect of AIH on neurophysiological measures, a two-way repeated-measures ANOVA was performed on raw single-pulse MEP amplitude data to test for differences between sessions and over time. A three-way repeated-measures ANOVA was performed on raw input/output curve data to determine whether MEP amplitude at the varying intensities differed between sessions and over time. Separate two-way repeated-measures ANOVAs were performed on the SICI and ICF ratios to determine whether SICI and ICF differed between sessions and over time. A three-way repeated-measures ANOVA was performed on SICF ratios to determine whether SICF at the two peaks differed between sessions and over time.

For all ANOVAs, Mauchley’s test of Sphericity was examined, and in the event of a violation of sphericity, the Greenhouse–Geisser correction was applied to adjust degrees of freedom. Effect sizes were described in terms of partial eta-squared (*η*_*p*_^2^, with *η*_*p*_^2^ < 0.06 representing a small effect, *η*_*p*_^2^ ≥ 0.06 a moderate effect, and *η*_*p*_^2^ ≥ 0.14 a large effect).

To assess the association between the extent of changes in SpO_2_ level and changes in TMS measures, Spearman’s correlations were performed to analyse the relationship between change in normalised SpO_2_ and change in MEP amplitude, SICI, ICF, and SICF (expressed as percentage change from pre) for AIH and NOR sessions separately.

## Results

### Arterial oxygen saturation (SpO_2_)

**Fig. 2:**
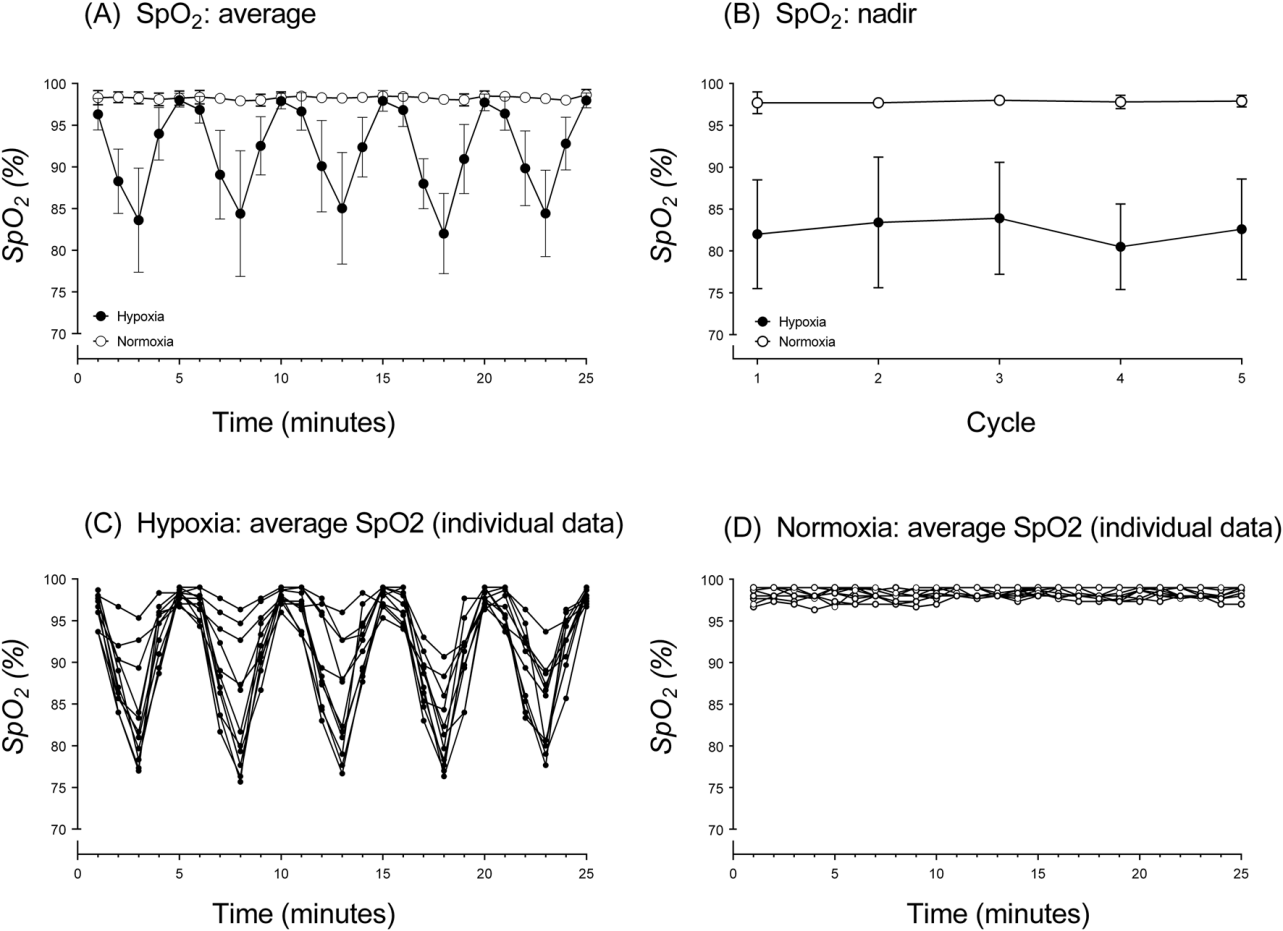
Mean ± SD oxygen saturation levels (% SpO_2_) for hypoxia and normoxia sessions. Group SpO_2_ data observed per minute over the 25 min of hypoxia and normoxia exposure (**A**). Group SpO_2_ nadir for each cycle for the 25 min of hypoxia and normoxia exposure (**B**). Individual SpO_2_ data from hypoxia protocol exposure per minute over 25 min of intervention (**C**). Individual SpO_2_ data from normoxia protocol exposure per minute over 25 min of intervention (**D**). Each data point reflects the average SpO_2_ level per minute and each line on the graph represents an individual participant

For the participants included in our analyses, there was a significant main effect of Session for the average SpO_2_ of the five 3-min hypoxic cycles (*F*_1,10_ = 98.75, *p* < 0.001, *η*_*p*_^2^ = 0.91), with significantly lower SpO_2_ values in AIH (mean ± SD: 89.8 ± 2.8%, range: 85.4–94.1%) compared with NOR (mean ± SD: 98.3 ± 0.5%, range: 97.6–99.0%). However, for the mean SpO_2_ for the hypoxic cycles, there was no significant main effect of Cycle (*F*_4,40_ = 0.68, *p* = 0.608, *η*_*p*_^2^ = 0.06) and no significant Session*Cycle interaction (*F*_4,40_ = 0.64, *p* = 0.637, *η*_*p*_^2^ = 0.06). For the nadir SpO_2_ data, there was a significant main effect of Session (F_1,10_ = 97.58, *p* < 0.001, *η*_*p*_^2^ = 0.91), with a significantly lower SpO_2_ in AIH than in NOR. The nadir in SpO_2_ across each cycle ranged from 76.2 to 90.2% (mean ± SD: 82.5 ± 4.9%). There was no significant main effect of Cycle (*F*_4,40_ = 0.86, *p* = 0.495, *η*_*p*_^2^ = 0.08) and no significant Session*Cycle interaction (*F*_4,40_ = 0.82, *p* = 0.519, *η*_*p*_^2^ = 0.08) for the nadir. There was a cyclical reduction in SpO_2_ levels during AIH but no significant change in SpO_2_ levels during NOR (Fig. 2). Mean SpO_2_ across the final 60 s of each hypoxic cycle ranged from 77.1% to 91.2% (mean ± SD: 83.9 ± 4.8%), which was similar to the results observed for the nadir.

### Single-pulse TMS measures

**Fig. 3:**
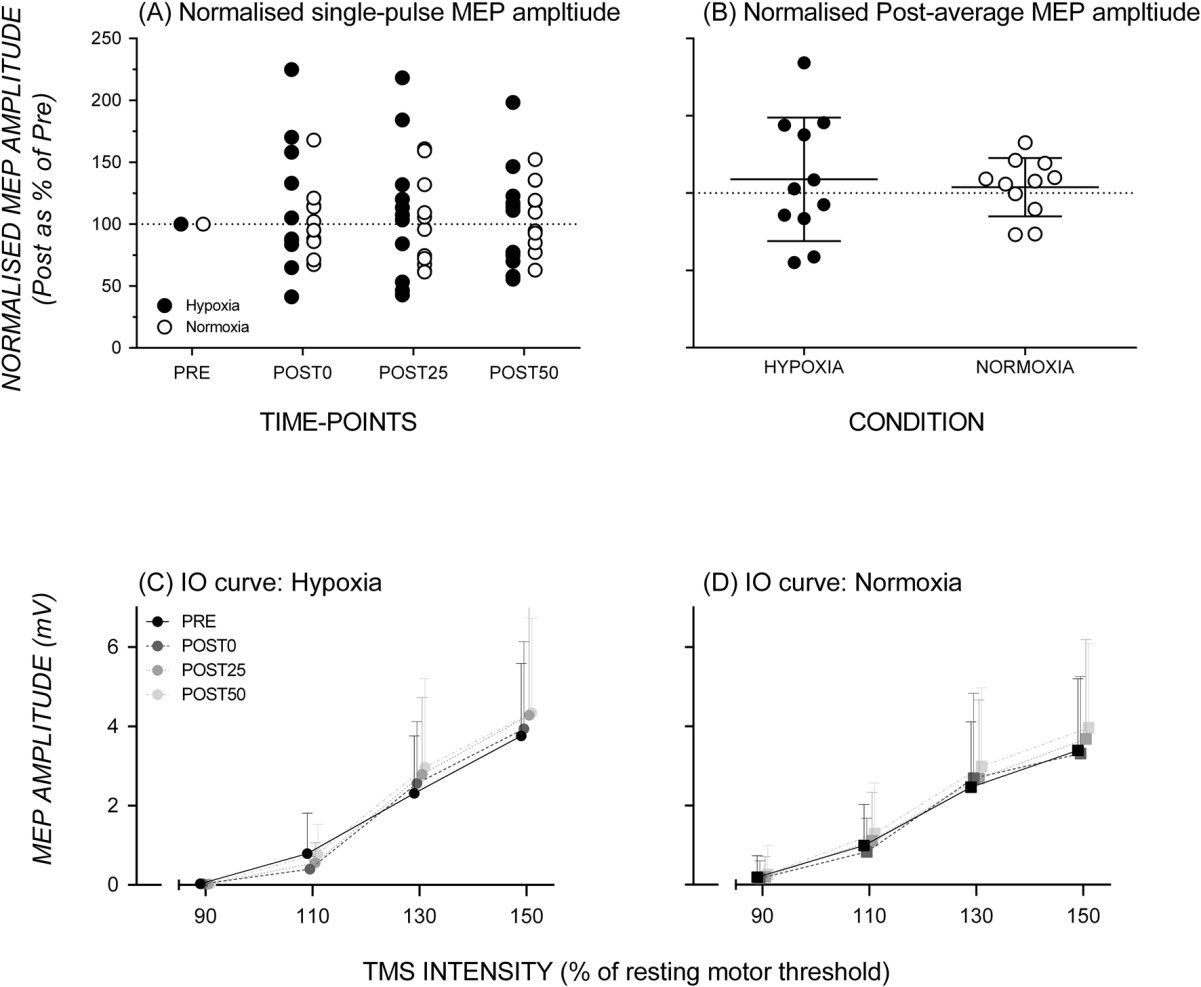
No change in Motor-Evoked Potentials (MEPs) by single-pulse TMS and in Input/Output curves after exposure to hypoxia compared to normoxia**.** Normalised MEP amplitude data observed before and after hypoxia (filled symbols) and normoxia (open symbols) exposure; each symbol reflects data from one individual (**A**). Column scatterplots of normalised MEP amplitude (post-time-points averaged and presented as a percentage of pre) data (*n* = 18) at baseline (dotted line) and after exposure to hypoxia and normoxia (**B**). Group MEP amplitude as a function of stimulation intensity hypoxia and normoxia sessions, respectively (**C**, **D**). Data points are off set horizontally for clarity in representation of data points

The baseline single-pulse MEP amplitude was 2.31 ± 1.46 mV for the AIH session and 1.94 ± 0.84 mV for the NOR session. A repeated-measures ANOVA performed on single-pulse MEP amplitude showed no significant main effect of Session (*F*_1,10_ = 0.67, *p* = 0.433, *η*_*p*_^2^ = 0.06) or Time (*F*_3,30_ = 0.14, *p* = 0.938, *η*_*p*_^2^ = 0.01), and no significant Session*Time interaction (*F*_3,30_ = 0.15, *p* = 0.932, *η*_*p*_^2^ = 0.01) (Fig. 3A, B).

### Input/output curve

For the AIH condition, the baseline MEP amplitude for the I/O curve was 0.03 ± 0.03 mV for 90%RMT, 0.79 ± 1.03 mV for 110%RMT, 2.31 ± 1.45 mV for 130%RMT, and 3.76 ± 1.73 mV for 150%RMT. For the NOR condition, the baseline MEP amplitude for the I/O curve was 0.19 ± 0.55 mV for 90%RMT, 0.99 ± 1.04 mV for 110%RMT, 2.46 ± 1.65 mV for 130%RMT, and 3.39 ± 1.81 mV for 150%RMT. As expected, the ANOVA showed a significant main effect of Intensity (*F*_1.1,11.2_ = 31.42, *p* < 0.000, *η*_*p*_^2^ = 0.76) but no significant main effect of Session (*F*_1,10_ = 0.26, *p* = 0.875, *η*_*p*_^2^ < 0.01) or Time (*F*_3,30_ = 2.42, *p* = 0.086, *η*_*p*_^2^ = 0.20). There was a significant Session*Intensity interaction (*F*_2.2,21.7_ = 4.23, *p* = 0.026, *η*_*p*_^2^ = 0.30). Post hoc analyses revealed that, for both AIH and NOR, MEP amplitude enlarged as TMS intensity increased from 90 to 150% of rMT, but remained unchanged across all time-points in AIH and NOR sessions (Fig. 3C, D). The significant Session*Intensity interaction was driven by the higher MEP amplitude in AIH relative to NOR at 150% MSO, which failed to reach the conventional significance level (*p* = 0.095). There were no other significant interactions: Session * Time (*F*_3,30_ = 0.09, *p* = 0.931, *η*_*p*_^2^ = 0.01); Time * Intensity (*F*_3.0,30.0_ = 1.26, *p* = 0.307, *η*_*p*_^2^ = 0.11); Session * Time * Intensity (*F*_4.7,46.9_ = 0.49, *p* = 0.774, *η*_*p*_^2^ = 0.05).

### Paired-pulse TMS measures

#### Short-interval intracortical inhibition (SICI)

**Fig. 4:**
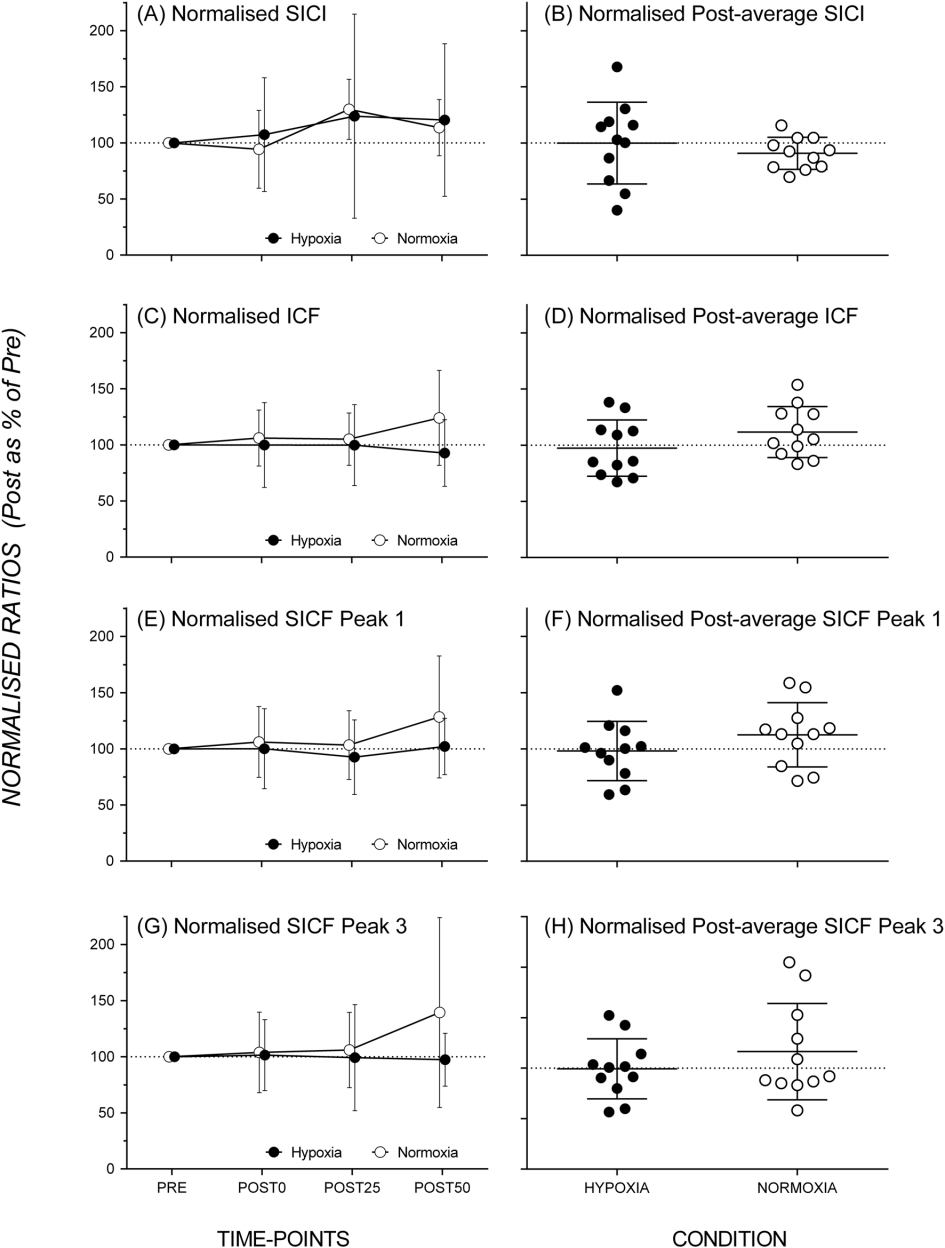
No change in intracortical inhibitory and facilitatory circuits after exposure to hypoxia compared to normoxia. The left column shows Normalised average SICI (**A**), ICF (**C**), SICF Peak 1 (**E**), and SICF Peak 3 (**G**) ratio (mean ± SD) data observed before and after hypoxia (filled symbol) and normoxia (open symbol) exposure. Data points are off-set horizontally for clarity in representation of data points. The right column shows scatterplots of normalised group SICI (**B**), ICF (**D**), SICF Peak 1 (**F**), and SICF Peak 3 (**H**) ratio (pre- as a percentage of post-average) data after exposure to hypoxia and normoxia

The baseline SICI ratio was 0.29 ± 0.17 for the AIH session and 0.33 ± 0.24 for the NOR session. The repeated-measures ANOVA showed no significant main effect of Session (*F*_1,10_ = 2.34, *p* = 0.157, *η*^2^ = 0.19), no significant main effect of Time (*F*_3,30_ = 2.29, *p* = 0.098, *η*_*p*_^2^ = 0.19), and no significant Session*Time interaction (*F*_3,51_ = 0.35, *p* = 0.789, *η*_*p*_^2^ = 0.03). SICI elicited by paired-pulse TMS did not differ after exposure to either AIH or NOR (Fig. 4).

#### Intracortical Facilitation (ICF)

The baseline ICF ratio was 1.52 ± 0.39 for the AIH session and 1.37 ± 0.32 for the NOR session. There was no significant main effect of Session (*F*_1,10_ = 0.17, *p* = 0.685, *η*_*p*_^2^ = 0.02) or Time (*F*_3,30_ = 0.30, *p* = 0.823, *η*_*p*_^2^ = 0.03), and there was no significant Session*Time interaction (*F*_3,30_ = 0.90, *p* = 0.452, *η*_*p*_^2^ = 0.08). As shown in Fig. 4, ICF elicited by paired-pulse TMS did not significantly differ across session or time-points.

#### Short-interval intracortical facilitation (SICF)

The baseline SICF ratio for the 1.5 ms ISI was 1.41 ± 0.52 for the hypoxia session and 1.37 ± 0.38 for the normoxia session. The baseline SICF ratio for the 4.5 ms ISI was 1.15 ± 0.30 for the hypoxia session and 1.11 ± 0.29 for the normoxia session. There was a significant main effect of Peak (*F*_1,10_ = 11.58, *p* = 0.007, *η*_*p*_^2^ = 0.54) but no significant main effect of Session (*F*_1,10_ = 0.96. *p* = 0.351, *η*_*p*_^2^ = 0.09) or Time (*F*_3,30_ = 2.24, *p* = 0.105, *η*_*p*_^2^ = 0.18). There were also no significant Session*Peak (*F*_1,10_ = 0.12, *p* = 0.734, *η*_*p*_^2^ = 0.01), Session*Time (*F*_3,30_ = 0.85, *p* = 0.479, *η*_*p*_^2^ = 0.08), Peak*Time (*F*_3,30_ = 0.26, *p* = 0.853, *η*_*p*_^2^ = 0.03), and Session*Peak*Time (*F*_3,30_ = 0.35, *p* = 0.793, *η*_*p*_^2^ = 0.03) interactions. SICF at both peaks (1.5 and 4.5 ms ISIs) did not differ significantly across sessions or time-points (Fig. 4).

### Associations between oxygen saturation and neurophysiological measures

Spearman’s bivariate correlations showed that there was no significant relationship between normalised SpO_2_ and changes in MEP amplitude, SICI, ICF, SICF Peak 1 (ISI 1.5 ms), or SICF Peak 3 (ISI 4.5 ms) (all *r* < 0.32, all *p* > 0.339).

## Discussion

This study aimed to characterise the effects of AIH on changes in corticospinal excitability, as well as the excitability of intracortical facilitatory and inhibitory circuits within M1. Results show that MEP amplitude and input/output curves did not change with AIH or NOR. Contrary to our hypothesis, these results suggest that exposure to hypoxia did not affect corticospinal excitability. In addition, neither SICI, ICF, nor SICF changed with AIH or NOR. This suggests, in agreement with our hypothesis, that exposure to hypoxia did not alter the excitability of intracortical inhibitory or facilitatory circuits. Our results do not align with those from Christiansen et al. (2018), as AIH failed to induce short-term neuroplastic adjustments in the corticospinal system. These findings, however, remain specific to the experimental parameters used (FiO_2_ = 10% for 3-min on and 2-min off, over 5 cycles in healthy young adults).

### Variable oxygen saturation levels during acute intermittent hypoxia exposure

As expected, AIH induced cyclical reductions in SpO_2_ levels. However, there was large inter-individual variability with minimum SpO_2_ values ranging from 76.2 to 90.2% (mean ± SD: 82.5 ± 4.9%). The fact that the same external stimulus (i.e., induced FiO_2_ levels in this study) initiates a different internal response (i.e., SpO_2_), presumably due to the variable nature of an individual’s oxyhaemoglobin dissociation curve, is a well-described phenomenon in the literature (Costello et al. 2020). This variability in the SpO_2_ response for a given FiO_2_ is known to increase with more severe levels of hypoxia, and it is therefore not surprising that substantial variability was observed using a FiO_2_ of 10%. In the literature, FiO_2_ levels ranging between 9 and 16% have mostly been used when administering AIH. Indeed, FiO_2_ levels below 9% are generally avoided as this is associated with greater risk for side effects such as hypoxic brain injury and cardiac arrhythmias (Navarrete-Opazo and Mitchell 2014). A large inter-individual variation in the degree of ventilatory drive in response to hypoxia may have increased variability in arterial hypoxemia (i.e., indirectly assessed here from SpO_2_ values) across participants. In the absence of direct minute ventilator measurement in our study, however, this remains speculative. Using a clamped SpO_2_, as opposed to a fixed FiO_2_ (i.e., as used in this study), variability in the response would have been decreased (Soo et al. 2020). Future research could also consider measuring an index of hyperventilation (e.g., partial pressure of end tidal carbon dioxide) to determine whether the ventilatory response is associated with changes in corticospinal excitability.

Compared to the present study, Christiansen et al. (2018) delivered slightly lower oxygen levels (FiO_2_ ~ 9.4%) during their AIH protocol composed of 15 cycles of 1-min on and 1-min off pattern for 30-min, ultimately totalling 15-min of hypoxic exposure (identical to the current study). While Christiansen et al. (2018) also showed large inter-individual variability in the reduction of SpO_2_ levels with hypoxic exposure, their SpO_2_ levels were lower across the 15-min of hypoxia (range: ~ 80–85%) than those observed for the 15-min exposure in our study (89.8 ± 2.8%, range: 85.4–94.1%). While we did observe lower SpO_2_ values in the final minute of each 3-min cycle (83.9 ± 4.8%), it is possible that the different cycle times we implemented (3-min hypoxia followed by 2-min normoxia) compared to that used by Christiansen et al. (2018) underpins these differences. Indeed, data presented by Christiansen et al. (2018) appear to show a trend for SpO_2_ to decrease across the first six 1-min cycles (between cycle analyses not reported), which may represent an accumulative effect of the hypoxic exposure with more frequent but shorter normoxic recovery periods, which we did not observe.

Although there was no association between change in SpO_2_ levels and change in MEP amplitude from baseline to post-exposure, it is possible that there is some interplay between SpO_2_ and plastic changes in the corticospinal system that might be observed with a larger sample. The relationship between changes in SpO_2_ levels and changes in MEP was not directly reported by Christiansen et al. (2018). However, the difference in results between the two studies suggests that a slightly more severe FiO_2_ level of 9.4% (used by Christiansen et al. 2018) compared to 10% used in the current study, as well as the more frequent yet briefer hypoxic exposures, might have resulted in larger changes in SpO_2_ levels which might have consequently evoked changes in TMS measures. This explanation is speculative and remains to be tested.

### Corticospinal excitability

There were no significant changes in single-pulse MEP amplitude or input/output curve following exposure to AIH, compared to normoxia, across all time-points. This result is inconsistent with the study by Christiansen et al. (2018), who did report increases in MEP amplitude following exposure to a 30-min AIH protocol. The lack of change in MEP amplitude at any of the tested TMS intensities suggests that neuronal recruitment patterns in M1 were not affected by AIH for the participants tested in our study.

### Intracortical circuit excitability

There were no significant changes in SICI and ICF following exposure to AIH across all time periods. SICI provides a measure of intracortical inhibition and likely reflects activation of GABA inhibitory circuits (Kujirai et al. 1993; Di Lazzaro et al. 2006), while ICF is suggested to be a facilitatory circuit modulated by glutamatergic mediated processes (Di Lazzaro and Ziemann, 2013). The lack of changes in SICI and ICF may suggest that AIH exposure did not cause changes in GABAergic-medicated intracortical inhibitory and glutamatergic intracortical facilitatory pathways within M1. Our study was the first to measure SICF in response to AIH. The results also showed that there were no differences in SICF between the AIH and NOR sessions, suggesting that the excitability of intracortical facilitatory circuits mediated by I-wave generating processes within M1 was not likely affected by AIH exposure (Stefan et al. 2004; Sale et al. 2007; Kamke et al. 2012, 2014).

Given the absence of any changes in intracortical processes in the current study, it is interesting to speculate on potential mechanisms of AIH-induced neuroplasticity. Studies examining the physiological mechanisms of hypoxia-induced neuroplasticity have largely focussed on SCI injury and on respiratory and spinal motoneurons (Fuller et al. 2000, 2003; Dale-Nagle et al. 2010; Gonzalez-Rothi et al. 2015a; Prosser-Loose et al. 2015). In SCI patients, the specific mechanisms of neuroplasticity are sensitive to the hypoxic dose. AIH activates serotonin-dependent mechanisms, known as respiratory long-term facilitation (Hayashi et al. 1993; Mitchell et al. 2001), which enhance ventilation or respiratory motor output through the release of serotonin that, in turn, strengthens synaptic pathways to phrenic motor neurons (Hayashi et al. 1993; Wilkerson and Mitchell 2009). Sustained or ‘severe’ doses of induced hypoxia activate adenosine-mediated pathways of long-term facilitation (Nichols et al. 2012; Devinney et al. 2016). Serotonin and adenosine, while both inducing long-term facilitation, are opposing mechanisms that could result in no net effect of inducing hypoxia. Given the severity of the hypoxia protocol affects these pathways, AIH dose is a key consideration for hypoxia research (Nichols et al. 2012).

### Limitations and future considerations

The current study recruited a larger proportion of female than male participants, whereas the Christiansen et al.’s (2018) study only recruited male participants. The inclusion of female subjects in our study, while critical for ensuring results that are generalizable within the population, requires further consideration. Menstrual cycle and menopause are known to affect corticospinal excitability (Zoghi et al. 2015), implying that the stage of the menstrual cycle should be controlled for or recorded in research studies. In the current investigation, female participants could have been at different stages of their menstrual cycle in the two experimental sessions. Furthermore, the sample size of the study was further reduced based on participants not demonstrating a substantial response to hypoxic exposure or based on pre-stimulus EMG which overall likely decreased the power of the study. The effect sizes associated with the Session * Time interaction for all measures are < 0.08 (*small*-to-*medium* effect size). Accordingly, the study would have required a larger sample size to detect significant differences.

MEP amplitude elicited by single-pulse TMS and I/O curves reflect excitability in both cortical and sub-cortical pathways including the spinal cord (Rothwell 1997, 2011; Ridding and Rothwell 2007; Rossini et al. 2015). While we did not observe changes in single-pulse TMS and I/O curve measures, these TMS measures may not have been as sensitive for identification of changes occurring specifically in the spinal cord. As shown by Christiansen et al. (2021), AIH can induce changes at the spinal level; future research should include targeted examination of spinal circuit excitability using techniques such as the Hoffman–Reflex and cervicomedullary motor-evoked potential (McNeil et al. 2013) to further characterise changes at the spinal level following AIH.

For future studies, a clamp-based approach to inducing hypoxia could be used whereby the external FiO_2_ levels are manipulated to evoke a desired SpO_2_ level in each participant. A desired level of hypoxia FiO_2_ may be considered at values less than 10% as prior literature on continuous or intermittent hypoxia exposure suggests that levels above 10% may not affect corticospinal excitability (Szubski et al*.* 2006; Goodall et al. 2010; Rupp et al. 2012; Christiansen et al. 2018). The pattern of exposure (i.e., the time of exposure to hypoxia and normoxia in each cycle and the number of cycles) must also be factored in when determining the optimal AIH dose. Currently, technological limitations prevent studies from being able to rapidly and accurately adjust the FiO_2_ levels in response to an individual’s SpO_2_ when inducing intermittent hypoxia; therefore, with future advances in technology, such manipulations and clamp-based studies may be warranted. Finally, in the current study, we did not ask participants to state which condition they thought they had in the two sessions to verify the effectiveness of our condition blinding strategy.

## Conclusions

Overall, AIH did not modify corticospinal excitability and excitability of GABAergic, glutamatergic, and I-wave generating processes acting within M1 when measured up to 50-min after the intervention. Our findings do not support using AIH to facilitate neuroplasticity, at least under present circumstances (FiO_2_ 10%). Future research systematically examining the effects of varying doses of AIH on cortical excitability is warranted given that AIH has been shown to improve function in patients with spinal cord injury.

## Supplementary Information

Below is the link to the electronic supplementary material.

Supplementary file1 (DOCX 221 KB)

## Abbreviations

AIH: Acute intermittent hypoxia
CNS: Central nervous system
EMG: Electromyography
FiO_2_: Fraction of inspired oxygen
ICF: Intracortical facilitation
MEP: Motor-evoked potential
NOR: Normoxia
SICF: Short-interval intracortical facilitation
SICI: Short-interval intracortical inhibition
TMS: Transcranial magnetic stimulation
