# Supplementary material for: Effects of acute intermittent hypoxia on corticospinal excitability within the primary motor cortex
Source: Eur J Appl Physiol. 2022 Jun 25;122(9):2111–23. doi: 10.1007/s00421-022-04982-8 (PMC9381468; doi:10.1007/s00421-022-04982-8)
Supplement: Supplementary file 1 — Supplementary file1 (DOCX 221 KB) [file 421_2022_4982_MOESM1_ESM.docx]

**Title:** Effects of Acute Intermittent Hypoxia on Corticospinal Excitability within the Primary Motor Cortex.

**Author Information:** Shivani Radia^1^, Ann-Maree Vallence^2^, Hakuei Fujiyama^2^, Rose Fitzpatrick^3^, Sarah Etherington^1^, Brendan R. Scott^2,3^, Olivier Girard^5^.

**Affiliations:**

1. Discipline of Medical, Molecular and Forensic Sciences, Murdoch University, Perth, Australia
2. Centre for Healthy Ageing, Murdoch University, Perth, Australia
3. School of Psychology and Sports Science, Murdoch University, Perth, Australia
4. Murdoch Applied Sports Science Laboratory, Discipline of Exercise Science, Murdoch University, Perth, Australia
5. School of Human Sciences (Sport Science, Exercise and Health), University of Western Australia, Perth, Australia

**Corresponding Author:** Olivier Girard – olivier.girard@uwa.edu.au

*Supplementary Table 1.* Repeated measures ANOVAs to test for differences between the two Pre measurements (Pre-1 and Pre-2) for TMS measures in the session testing Acute Intermittent Hypoxia (AIH) and the session testing normoxia (NOR).

| **Data** | **F** | **d.f** | **P** | **Effect size** |
| --- | --- | --- | --- | --- |
| Single pulse MEP |  |  |  |  |
| Session | 1.415 | 1,10 | 0.262 | 0.124 |
| Pre | 0.266 | 1,10 | 0.617 | 0.026 |
| Session * Pre | 0.562 | 1,10 | 0.471 | 0.053 |
| SICI |  |  |  |  |
| Session | 0.752 | 1,10 | 0.406 | 0.070 |
| Baseline | 0.498 | 1,10 | 0.496 | 0.047 |
| Session * Baseline | 2.262 | 1,10 | 0.163 | 0.184 |
| ICF |  |  |  |  |
| Session | 0.581 | 1,10 | 0.463 | 0.055 |
| Baseline | 1.443 | 1,10 | 0.257 | 0.126 |
| Session * Baseline | 1.329 | 1,10 | 0.276 | 1.329 |
| SICF ISI 1.5ms |  |  |  |  |
| Session | 0.001 | 1,10 | 0.997 | 0.000 |
| Baseline | 0.620 | 1,10 | 0.449 | 0.058 |
| Session * Baseline | 0.361 | 1,10 | 0.561 | 0.035 |
| SICF ISI 4.5ms |  |  |  |  |
| Session | 0.652 | 1,10 | 0.438 | 0.061 |
| Baseline | 1.612 | 1,10 | 0.233 | 0.139 |
| Session * Baseline | 0.001 | 1,10 | 0.977 | 0.001 |

*Supplementary Table 2.* Repeated measures ANOVA to test for differences between the Pre input/output curves in the session testing Acute Intermittent Hypoxia (AIH) and the session testing normoxia (NOR).

| **Data** | **F** | **d.f** | **P** | **Effect size** |
| --- | --- | --- | --- | --- |
| Input/output curves | | | | |
| Session | 0.269 | 1,10 | 0.615 | 0.026 |
| Intensity | 41.101 | 1.4,14.4 | <0.001 | 0.804 |
| Session * Intensity | 0.575 | 3,30 | 0.636 | 0.054 |


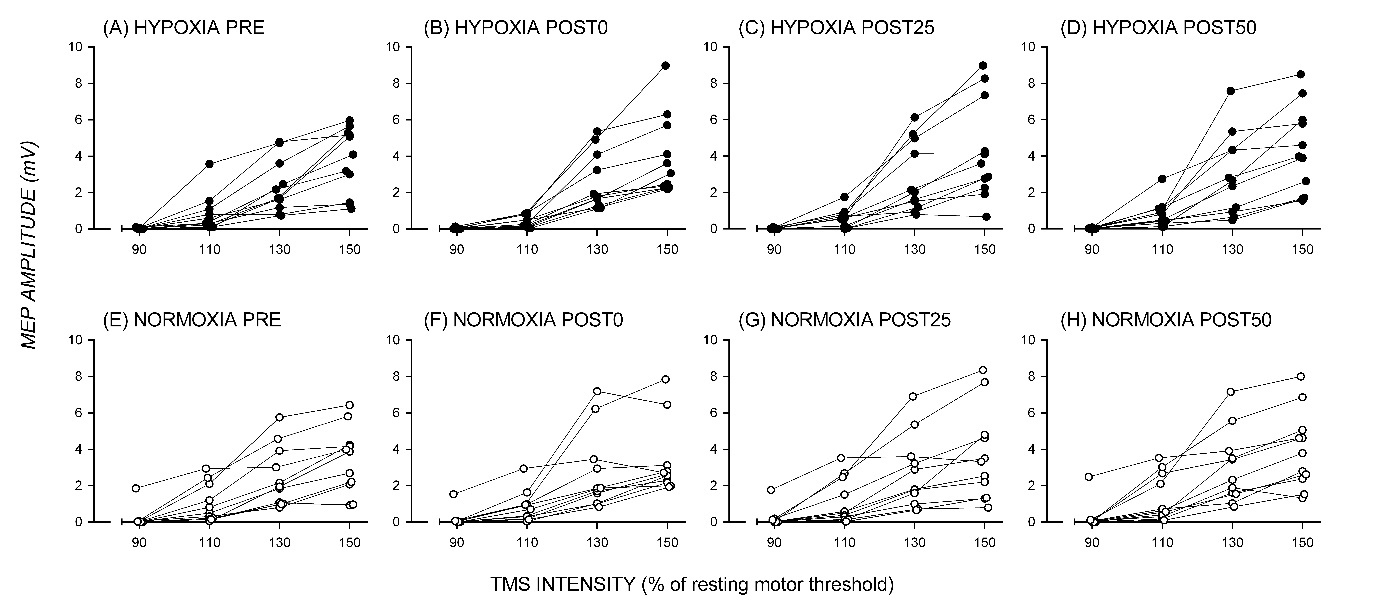


*Supplementary Figure 1.* Input/Output curves after exposure to hypoxia compared to normoxia. All panels show individual MEP amplitude as a function of stimulation intensity: panels A-D hypoxia; panels E-H normoxia sessions. Some data points are off-set horizontally for clarity.
